# Lycopene prevents carcinogen-induced cutaneous tumor by enhancing activation of the Nrf2 pathway through p62-triggered autophagic Keap1 degradation

**DOI:** 10.18632/aging.103132

**Published:** 2020-05-04

**Authors:** Siliang Wang, Yuan-Yuan Wu, Xu Wang, Peiliang Shen, Qi Jia, Suyun Yu, Yuan Wang, Xiaoman Li, Wenxing Chen, Aiyun Wang, Yin Lu

**Affiliations:** 1 Jiangsu Key Laboratory for Pharmacology and Safety Evaluation of Chinese Materia Medica, School of Pharmacy, Nanjing University of Chinese Medicine, Nanjing 210023, P.R. China; 2 Jiangsu Collaborative Innovation Center of Traditional Chinese Medicine (TCM) Prevention and Treatment of Tumor, Nanjing University of Chinese Medicine, Nanjing 210023, P.R. China; 3 Nanjing Drum Tower Hospital, The Affiliated Hospital of Nanjing University Medical School, Nanjing 210008, P.R. China

**Keywords:** lycopene, cutaneous tumor, autophagy, Nrf2, chemoprevention

## Abstract

Biologically active natural products have been used for the chemoprevention of cutaneous tumors. Lycopene is the main active phytochemical in tomatoes. We herein aimed to assess the cancer preventive effects of lycopene and to find potential molecular targets. In chemically-induced cutaneous tumor mice and cell models, lycopene attenuated cutaneous tumor incidence and multiplicity as well as the tumorigenesis of normal cutaneous cells in phase-selectivity (only in the promotion phase) manners. By utilizing a comprehensive approach combining bioinformatics with network pharmacology, we predicted that intracellular autophagy and redox status were associated with lycopene’s preventive effect on cutaneous tumors. Lycopene stimulated the activation of antioxidant enzymes and the translocation of the transcription factor Nrf2 (nuclear factor erythroid 2-related factor 2) that predominantly maintained intracellular redox equilibrium. The cancer chemopreventive effects were mediated by Nrf2. Further, lycopene enhanced the expression of autophagy protein p62. Therefore this led to the degradation of Keap1(Kelch ECH associating protein 1), the main protein locking Nrf2 in cytoplasm. In conclusion, our study provides preclinical evidence of the chemopreventive effects of lycopene on cutaneous tumors and reveals the mechanistic link between lycopene’s stimulation of Nrf2 signaling pathway and p62-mediated degradation of Keap1 via the autophagy-lysosomal pathway.

## INTRODUCTION

As one of the most common cancers worldwide, cutaneous carcinoma has over one million new cases each year, with annual cost for treatment surpassing 8 billion dollars [1, 2]. Nevertheless, it still cannot be effectively treated in regards to both poor clinical outcomes and out-of-pocket expenditure [3, 4]. In an economical, non-toxic, and easily available manner, cancer chemoprevention uses substances to decelerate or to eliminate the progression of intraepithelial precancerous or neoplastic lesions to tumors. Therefore, cancer chemoprevention therapy has potential economic interests for cutaneous carcinoma and other types with skyrocketing morbidity [5, 6].

Epidemiological evidence supports an association between tomato consumption and reduced risk of prostate cancer. Clinical data suggests that continued consumption of tomato paste can alleviate UV-induced sunburn in humans. Lycopene, the primary phytochemical in tomatoes, has been reported to reduce the risks of many diseases [7, 8], with well-demonstrated anti-inflammatory, antimicrobial, and anti-aging activities [9, 10]. However, the preventive effects of this compound applied topically and the underlying mechanisms remain unclear. To this end, we herein utilized two models of chemically induced tumorigenesis to evaluate the chemopreventive effects of lycopene in vivo and in vitro.

Aggravated redox imbalance may originate from a series of carcinogens and lead to genetic mutation, genomic instability, and neoplastic transformation [11, 12]. The skin is the largest organ enduring external harmful substances, and is thus prone to carcinogenesis. Generally, by inducing sophisticated anti-stress defense responses, normal cutaneous cells adapt to adverse conditions in response to emergencies such as detrimental stress [13, 14]. Carcinogen-induced over-consumption or dysregulation of these processes is related with the pathogenesis of human cancers [15–17]. Therefore, we focused on lycopene-evoked defense mechanism to combat the carcinogens, which might clue the mechanisms that mediate the action of this compound.

Of the main mechanisms for cellular defense against electrophilic and oxidative stresses, the Keap1-Nrf2 system plays a paramount role in preventing cutaneous carcinoma and many other types [18, 19]. On this basis, we used Nrf2-/- mice and Nrf2KD cells to explore the relationship between the preventive effect of lycopene and the Nrf2 signaling pathway and related anti-oxidant system.

Selective autophagy is another important host defense mechanism [20]. Cytoplasmic materials are engulfed by autophagosomes through this bulk-degradation protective system. This system appears constitutively and is induced responding to intracellular stresses to maintain homeostasis by degrading toxic or unnecessary organelles, proteins and, pathogens [21, 22]. p62 protein plays a vital role in selective autophagy, generally acting as a cargo receptor for ubiquitinated substrates that fuse with lysosomes, resulting in substrate degradation [23]. Recently, p62 has been verified to participate in the Nrf2-Keap1 pathway through interacting with the Nrf2- binding site of Keap1 and inhibiting Keap1-Nrf2 interaction competitively, inducing the expressions of gene-encoding antioxidant enzymes [24]. In this study, we identified the endogenous interaction between Keap1 and p62 as well as the degradation of Keap1 in an autophagy-lysosomal manner after treatment with lycopene, which protected Nrf2 from Keap1-induced proteasomal degradation. These findings highlight the significance of lycopene in preventing cutaneous carcinoma, as a skin care supplement, can feasibly reduce the risk of this cancer, through a selective autophagy-controlled Nrf2 pathway activation manner.

## RESULTS

### Lycopene exerted preventive effects on chemically induced cutaneous tumor only in the promotion phase

**Figure 1:**
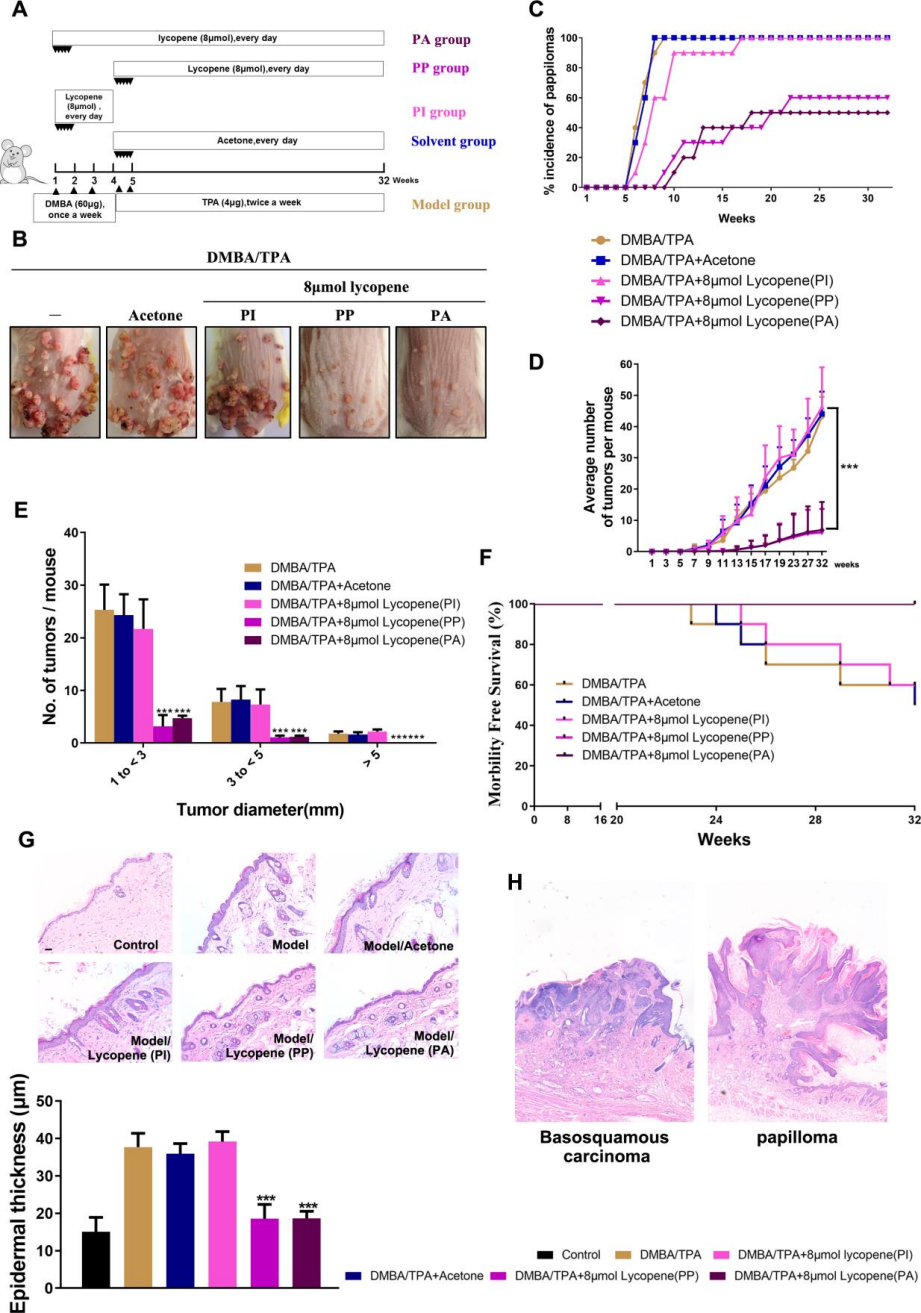
**Chemopreventive effect of lycopene on DMBA/TPA-induced cutaneous papilloma in vivo.** (**A**) The workflow of animals study as described in results and methods. (**B**) Representative images of papillomas in the indicated groups. (**C**) The incidence of papillomas in different treatment groups (n=10). (**D**) The average numbers of papillomas per mouse in the indicated groups (n=10). The data are presented as the mean ± SD. ***p < 0.001 (versus DMBA/TPA) (**E**) The average numbers of papillomas per mouse in different tumor diameter groups (n=10). The data are presented as the mean ± SD. ***p < 0.001 (versus DMBA/TPA). (**F**) Survival rates of mice in different treatment groups within 32 weeks. (**G**) Presentative images of epidermal proliferation and hyperplasia in the indicated groups (40×). (Bottom) Quantitative analysis of epidermal thickness based on H&E images (n=6). The data are presented as the mean ± SD. ***p < 0.001 (versus DMBA/TPA). (**H**) (Left panel) Basosquamous carcinoma was observed in mouse in DMBA/TPA model group, and local invasion of basosquamous carcinoma cells can be detected into stroma (40×). (Right panel) Only the benign papillomas can be found in PA and PP mouse with intact basement membranes and hyperplasia of the overlying epidermis (40×).

A two-stage 7, 12-dimethylbenzanthracene (DMBA)/ 12-O-tetradecanoylphorbol-13-acetate (TPA)-induced model, which has been used in our group and other labs for years as a successful mouse model of cutaneous papilloma, was first employed [17] (Figure 1A). Being organ-specific, it captures well-defined pathological progression from normal tissue to papilloma and finally squamous cell carcinoma (SCC) in human body. This model simulates the multi-step processes (mostly initiation and promotion) of cutaneous carcinogenesis, in which the initiation phase is mediated by DMBA and the promotion phase is induced by TPA [25].

To analyze the roles of lycopene in the two phases, we divided mice into four groups including three treatment groups and one model group with indicated chemical treatment at different stages (Figure 1A). One treatment group (PI group: pretreatment only before initiation) was administered by lycopene before DMBA-mediated initiation until TPA-initiated promotion. Similar to most cancer chemopreventive studies, another treatment group (PP group: pretreatment only before promotion) was administered between initiation and promotion until the end of this study. The third treatment group (PA group: pretreatment in all phases) was administered before DMBA-mediated initiation until the end of this study. With this grouping method, the preventive effects of lycopene on initiation phase, promotion phase and the whole process were assessed respectively.

As shown in Figure 1B, all the DMBA/TPA-treated mice have cutaneous papillomas on dorsal skins. Compared with the model group, pretreatment with lycopene markedly attenuated both the incidence rate (Figure 1C) and multiplicity (Figure 1D) of cutaneous papillomas only in PP and PA groups, and delayed the latency substantially from 5 to 8 weeks. Besides, the inhibitory effects of lycopene on tumorigenesis were proven by the distribution of papilloma sizes (Figure 1E). Furthermore, the survival rate of the model group plummeted compared with those of PP and PA groups. In the end, only 50% of mice in the model group remained alive, whereas almost all the mice in PP and PA groups survived bearing cutaneous papilloma (Figure 1F). Surprisingly, PP and PA groups had similar cutaneous papilloma incidence rates, multiplicities, latencies, and survival rates, suggesting lycopene exerted evident preventive effects on cutaneous papilloma only in the promotion phase.

Histological analysis further signified that lycopene notably attenuated the increased epidermal thickness (hyperplasia) induced by DMBA/TPA (Figure 1G). Furthermore, basosquamous carcinoma was observed in mouse in model group, and local invasion of basosquamous carcinoma cells can be detected in stroma. However, on the contrary, only the benign papillomas can be found in PA and PP mouse with intact basement membranes and hyperplasia of the overlying epidermis (Figure 1H).

**Figure 2:**
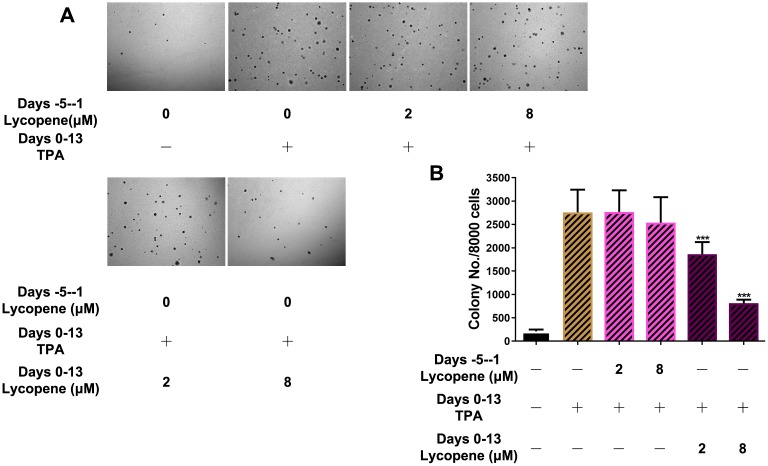
**Inhibitory effects of lycopene on TPA-induced transformation of JB6 P+ cells in vitro.** (**A**) Soft agar assay of cells with indicated treatment and time. Images were taken and analyzed using the ZEN pro 2012 imaging software on a Zeiss invert microscope under 100-fold magnification. (**B**) Quantitative analysis of the soft agar assay (n=3). The data are presented as the mean ± SD. ***p < 0.001 (versus TPA alone).

To further demonstrate the preventive effects of lycopene on cutaneous tumors in the promotion phase, we observed the anchorage-independent growth of JB6 P+ cells in soft agar induced by TPA (Figure 2). JB6 P+ cells were pretreated for 5 days with lycopene and then incubated with TPA in the presence or absence of this compound for another 14 days to induce malignant transformation. Pretreatment with 2 or 8 μM lycopene significantly inhibited the colony formation of JB6 P+ cells induced with TPA by about 45% and 71% (P < 0.001), respectively. When JB6 P+ cells were incubated in soft agar containing TPA without lycopene for another 14 days, pretreatment with lycopene at both concentrations hardly decreased the colony number (P > 0.05). In short, lycopene had potential chemopreventive effects on carcinogen-induced cutaneous tumor in the promotion phase.

### Predicted mechanism for the role of lycopene in the promotion phase of cutaneous tumor

**Figure 3:**
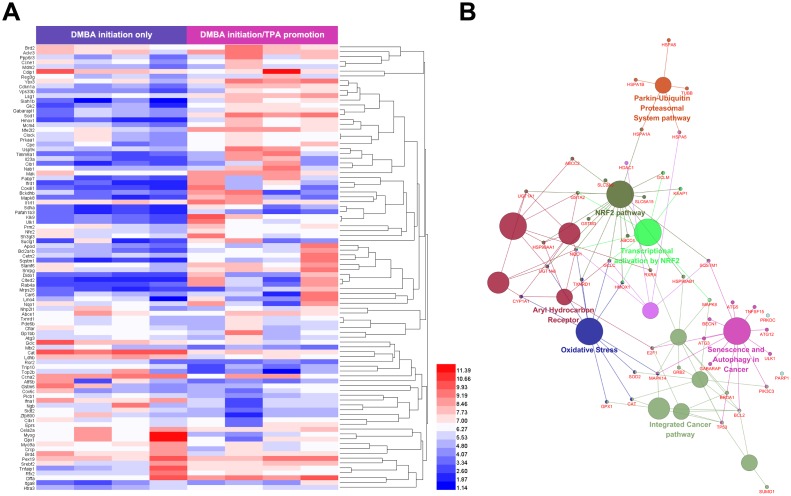
**Predicted mechanism for the role of lycopene in the promotion phase of cutaneous tumor.** (**A**) Identification of cutaneous tumor related targets by existing microarray data. 92 different expression genes identified were highly related to promotion phase of cutaneous tumor. Upper color bar represents sample classes. P< 0.05, FC>1.5 was considered as the cutoff value. (**B**) ClueGO pathway analysis of the candidate lycopene targets. Functionally grouped network of enriched categories was generated for the target genes. GO terms are represented as nodes, and the node size represents the term enrichment significance. Functionally related groups partially overlap. Only the most significant term in the group was labeled. Representative enriched pathway (P<0.05) interactions among candidate lycopene targets.

To explore the mechanism for the role of lycopene without blind searching, we utilized a comprehensive method combining bioinformatics, pharmacophore mapping, and network analysis. First, we predicted the putative targets of lycopene by using pharmacophore mapping, a web server for identifying the potential targets of active compounds [26]. According to the fit scores, top 100 putative targets were selected and sorted (Submission ID 160927141728, Supplementary Table 1). In general, compounds for use are determined by the functions of their affected targets that play crucial roles in pathological changes. Therefore, it is equally important to find the key joints of promotion phase of cutaneous tumor. Based on gene expression profiles, we used microarray data available in Arrayexpress (E-MEXP-188) to obtain the preventive signature of promotion phase [27]. In brief, one set of comparison were conducted. DMBA-initiated skin (D) was compared with DMBA-initiated, TPA-promoted skin (DT). This comparison produced a mixed response to the gene expression changes related with the cancer-promoting effects of TPA only. Differentially expressed genes with higher changes (P< 0.05, Fold Chang (FC)> 1.5) were considered as significantly altered merely in the promotion phase (Figure 3A). Based on this, networks in which the promotion-related preventive signature interacted with the putative targets of lycopene in a PPI (protein-protein interaction) manner were constructed [28]. Afterwards, the most important targets (candidate targets) were screened by utilizing topological features with a widely used plugin CytoNCA [29] (Supplementary Table 2). The interrelations between functional groups and their significances in biological networks were investigated by enrichment analysis to study the possible roles of these key targets [30]. As displayed in Figure 3B, the targets with topological features are significantly related with intracellular oxidative stress, Nrf2 pathway, and autophagy. This prediction was validated by subsequent experiments.

### Lycopene reversed the intracellular redox imbalance induced by carcinogens in vivo and in vitro

**Figure 4:**
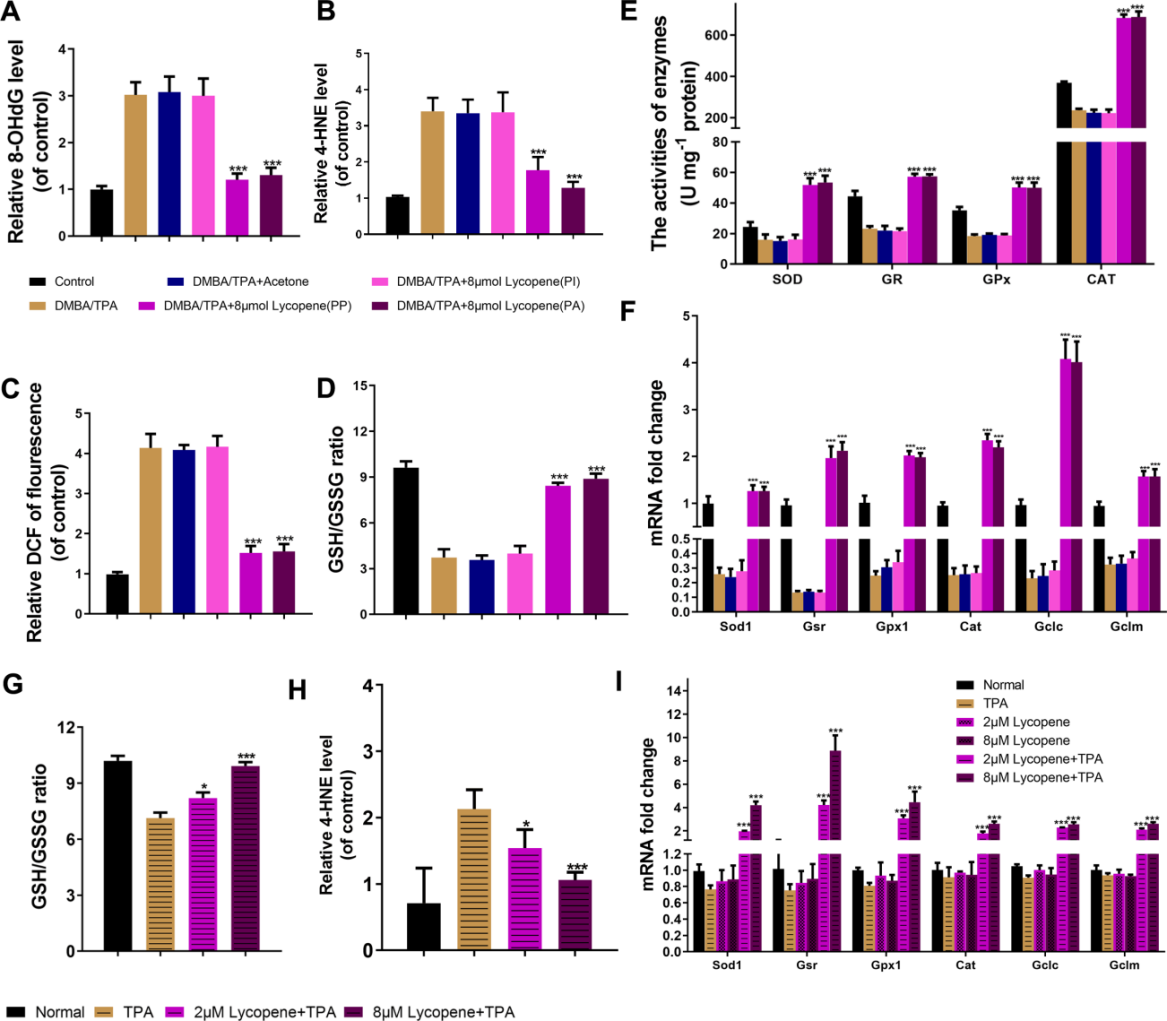
**Lycopene reversed the intracellular redox imbalance induced by carcinogens in vivo and in vitro.** (**A**, **B**) The level of 8-OhdG, 4-HNE in mouse skin of the indicated groups (n=3). (**C**) The DCFH-DA staining was used to detect ROS production in the indicated groups (n=3). (**D**) The GSH/GSSG ratio in mouse skin of the indicated groups (n=3). (**E**) The activities of SOD, GR, GPx and CAT in mouse skin of the indicated groups (n=3). (**F**) Total mRNA was isolated and analyzed to determine the levels of cat, sod1, gpx1, gsr, gclc and gclm expression using real-time qPCR in mouse skin of the indicated groups (n=3). (**G**) The effect of lycopene on GSH/GSSG ratio in lycopene-pretreated JB6 P+ cells with TPA stimulation (n=3). (**H**) The effect of lycopene on level of 4-HNE in vitro with TPA stimulation (n=3). (**I**) The mRNA levels of cat, sod1, gpx1, gsr, gclc and gclm were detected by real-time qPCR in lycopene-pretreated JB6 P+ cells with TPA stimulation (n=3). House-keeping gene gapdh was used as internal control. The data are presented as the mean ± SD. *p < 0.05, **p < 0.01 and ***p < 0.001 (versus DMBA/TPA or TPA).

As a commonly used tumor promoter, TPA can dramatically accelerate DMBA-induced malignant transformation of preneoplastic cell by inducing redox imbalance [31]. Therefore, we first explored whether this stress was reversed by lycopene pretreatment. As exhibited in Figure 4A–4C, lycopene significantly suppresses all the oxidative damages to lipid (4-HNE) and DNA (8-OHdG) compared to those of the model group in vivo and in vitro, in agreement with the results of ROS accumulation. Lycopene also rebalanced the GSH/GSSG ratio, partly representing the cellular redox condition commendably [32] (Figure 4D). The antioxidant defense enzyme system, including catalase (CAT), glutathione reductase (GR), superoxide dismutase (SOD), and glutathione peroxidase (GPx), essentially maintains cellular redox homeostasis [33]. Accordingly, whether lycopene triggered enzymatic response in the presence of TPA was further studied. As expected, the lower activities of these enzymes were reversed by this compound (Figure 4E). The mRNA levels of GSH and these antioxidant substances were also up-regulated significantly by lycopene pretreatment (Figure 4F). On these basis, the effect of lycopene on this antioxidant cell defence system has also been demonstrated in vitro, additionally (Figure 4G–4I), suggesting this compound prevented cutaneous tumor probably by maintaining intracellular redox homeostasis through regulating the antioxidant defense system.

### Lycopene activated the Nrf2 pathway in the presence of carcinogens in vivo and in vitro

**Figure 5:**
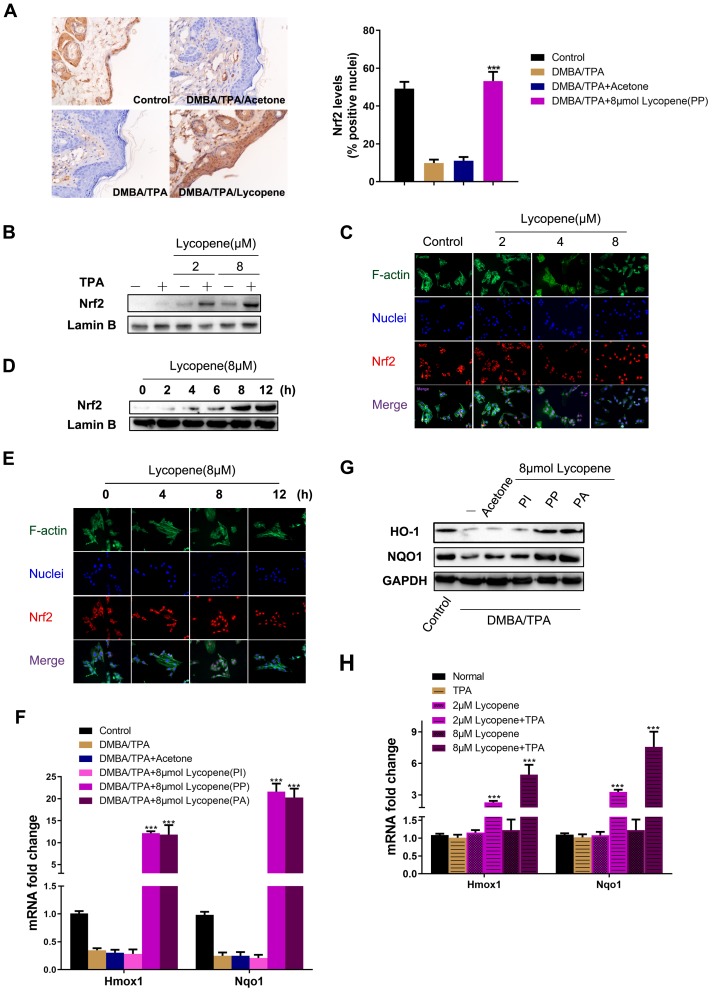
**Lycopene activated the Nrf2 pathway in the presence of carcinogens in vivo and in vitro.** (**A**) (Left panel) Representative images of Nrf2 immunohistochemistry staining in mouse epidermis in different groups (magnification 100×). (Right panel) Quantitative analysis of Nrf2 IHC results in left panel (n=9 per group). The data are presented as the mean ± SD. ***p < 0.001 (versus DMBA/TPA). (**B**) JB6 P+ cells were pretreated with increasing doses of lycopene for 12 hours and then exposed with or without TPA for additional 2 hours, and the nuclear levels of Nrf2 and LaminB1 were measured by Western blot. LaminB1 was used as the loading control. (**C**) JB6 P+ cells were pretreated with increasing doses of lycopene for 12 hours and then exposed with 20 ng/ml TPA for additional 2 hours. The immunofluorescence staining of Nrf2 was conducted as described in Materials and Methods (blue: nuclei, red: Nrf2, green: F-actin). (**D**) JB6 P+ cells were pretreated with 8 μM lycopene for various times and then exposed with TPA for additional 2 hours, and the nuclear levels of Nrf2 and LaminB1 were measured by Western blot. (**E**) The immunofluorescence staining of Nrf2. Treatment similar to (**D**). (**F**) Quantitative RT-PCR analysis of Nrf2 target genes in the mouse skin of the indicated groups (n=3). The data are presented as the mean ± SD. ***p < 0.001 (versus DMBA/TPA). (**G**) The levels of HO-1 and NQO1 in mouse skin were measured by Western blot. The results were representative of three independent experiments. (**H**) Treatment similar to (**B**), and mRNA levels of Hmox1 and Nqo1 were detected by real-time qPCR. GAPDH was used as the loading control. The data are presented as the mean ± SD. ***p < 0.001 (versus TPA alone).

Considering the vital role of Nrf2 in controlling redox status and in previous enrichment analysis results [34] (Figure 3B), we examined whether the Nrf2 pathway was activated by lycopene. Besides obviously inducing nuclear Nrf2 accumulation in the epidermis (Figure 5A), lycopene also increased nuclear Nrf2 localization in JB6 P+ cells relative to dosage and time (Figure 5B–5E). Subsequently, the transcription and translation of NQO1 and HO-1, two Nrf2 specific target genes, were coordinately up-regulated by lycopene in cutaneous tissue (Figure 5F, 5G). Then JB6 P+ cells were treated by different doses of lycopene without or with TPA, and NQO1 and HO-1 expressions were detected by qPCR. Lycopene pretreatment induced mRNA level of these two genes dose-dependently (Figure 5H) with TPA present. As evidenced by Figures 4 and 5, lycopene indeed enhanced the ability of intracellular defense system to electrophilically and oxidatively, activate the Nrf2 pathway, and effects of which can be dramatically enhanced with TPA present.

### Nrf2 was required for lycopene-induced prevention against cutaneous tumor

**Figure 6:**
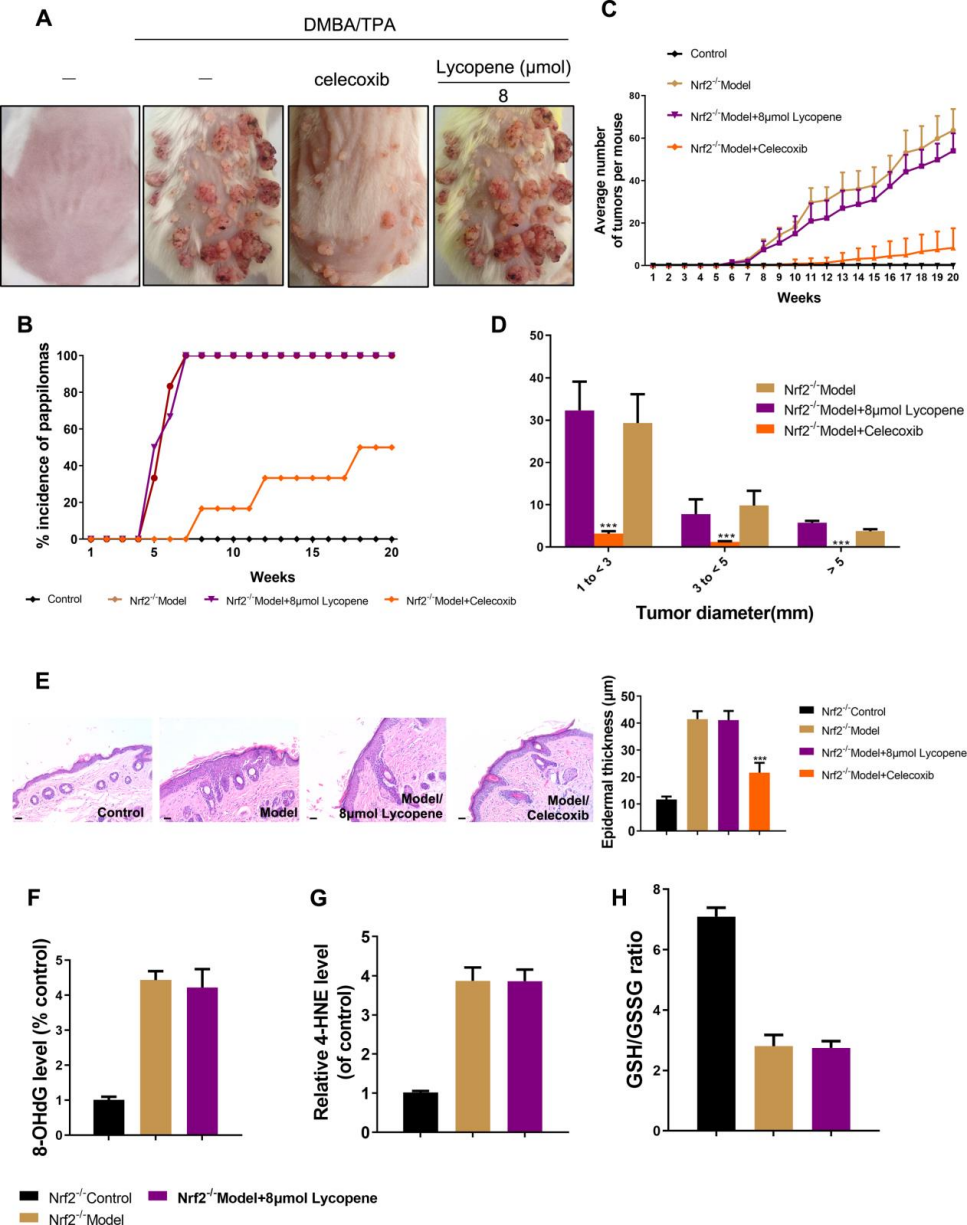
**Nrf2 was required for lycopene-induced prevention against cutaneous papilloma.** (**A**) Nrf2-/- animals were treated as previous study. Physical appearance of representatives of the indicated groups. (**B**) The incidence of papillomas in different treatment groups (n=6). (**C**) Average numbers of papillomas per mouse from the indicated groups (n=6). (**D**) Average numbers of papillomas per mouse in different tumor diameter groups (n=6). (**E**) Representative H&E staining images of mouse skin in different groups (40×). (Bottom) Quantitative analysis of epidermal thickness in H&E images (n=4). (**F**, **G**) The level of 8-OHdG, 4-HNE in mouse skin tissues of the indicated groups (n=4). (**H**) The GSH/GSSG ratio in mouse skin tissues of the indicated groups (n=4). The data are presented as the mean ± SD. ***p < 0.001 (versus DMBA/TPA).

To study whether Nrf2 was dominantly involved in the influence of lycopene on the promotion phase, cutaneous papilloma was induced in mice by global deletion of Nrf2 using DMBA/TPA. Interestingly, the preventive effects of lycopene on the incidence, multiplicity, and size of cutaneous papilloma induced by DMBA/TPA were reversed considerably (Figure 6A–6D). Moreover, there was no significant change of epidermal thickness in the DMBA/TPA-treated Nrf2-/- group and lycopene-treated group (Figure 6E). The redox imbalance status influenced by the carcinogens in Nrf2 knock-out mice also did not change with lycopene pretreated (Figure 6F–6H). Additionally, we evaluated the influence of lycopene on the malignant transformation of JB6 P+ cells after knock-down by using Nrf2 shRNA. Knock-down of Nrf2 facilitated the growth of JB6-shNrf2 cells in soft agar significantly compared with that of JB6-shMock cells (P < 0.01) (Supplementary Figure 1). Moreover, pretreatment with 2-8 μM lycopene significantly suppressed the anchorage-independent growth of JB6-shMock cells induced with TPA. Contrarily, this inhibition was attenuated in JB6-shNrf2 cells. Hence, Nrf2 played a critical role in lycopene-induced prevention against mouse cutaneous tumors due to carcinogen.

### Lycopene induced activation of Nrf2 by reducing Keap1 protein at the posttranslational level via the autophagy-lysosomal pathway

**Figure 7:**
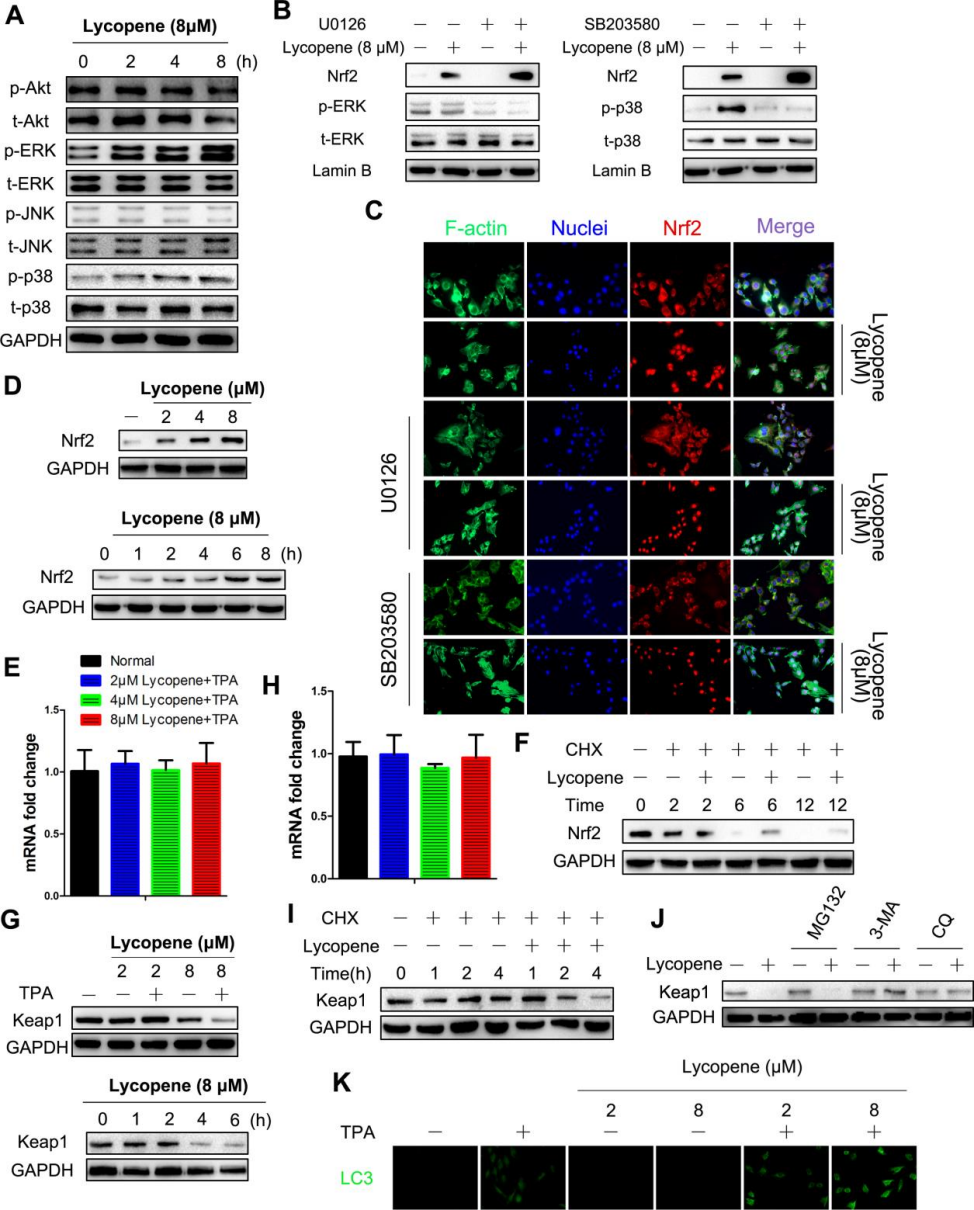
**Lycopene induced activation of Nrf2 by reducing Keap1 protein at the posttranslational level via the autophagy-lysosomal pathway.** (**A**) Cells were incubated with 8 μΜ lycopene for the indicated time and then exposed with TPA for additional 2 hours, and the expression of both phosphorylated and total forms of Akt, ERK1/2, p38 and JNK1/2 were measured by western blot analysis. (**B**) Cells were pre-treated with U0126 (left) or SB203580 (right), followed by lycopene treatment for 8 h. The nuclear protein extract was subjected to immunoblot analysis for the detection of Nrf2 expression. (**C**) Immunofluorescence analysis of Nrf2 was carried out as described in Methods. Treatment was similar to (**B**). (**D**) Time-dependent (bottom) and dose-dependent (top) study of lycopene on Nrf2 protein levels. Cells were pretreated with 8 lycopene for different times or with different doses of lycopene for 12 h and then exposed with TPA for additional 2 hours, and the Nrf2 protein level was assayed by Western blot. (**E**) Total RNA was isolated and analyzed to determine the levels of Nfe2l2 mRNA using real-time qPCR after lycopene treatment for 12 hours. House-keeping gene gaphd was used as the internal control. The data are presented as the mean ± SD. (n=3). (**F**) Cells were pretreated with CHX (0.5 g/ml) alone or in the presence of lycopene (8 μM) and TPA for various times. Nrf2 protein was examined by Western blot. (**G**) Time-dependent (right) and dose-dependent (left) study of lycopene on keap1 protein levels. Cells were pretreated with 8 μM lycopene for different times or with different doses of lycopene for 4 h and then exposed with TPA for additional 2 hours, and the keap1 protein level was assayed by western blot. (**H**) Total RNA was isolated and analyzed to determine the levels of Keap1 expression using real-time qPCR after lycopene treatment for 6 hours. House-keeping gene gapdh was used as the control. The data are presented as the mean ± SD. (n=3). (I) Cells were pretreated with CHX (0.5 g/ml) alone or in the presence of lycopene (8 μM) and TPA for various times. keap1 protein was examined by Western blot. (**J**) Cells were treated with MG132 (1 μM) or 3-MA (1 mM) or CQ (25 μM) for 1 h. Lycopene (8 μM) was added to cells for 6 hours, and then exposed with TPA for additional 2 hours. Expression of keap1 protein was examined by western blot. (**K**) Dose-dependent study of lycopene on LC3 protein levels. Cells were pretreated with different doses of lycopene for 6 h and then exposed with or without TPA for additional 2 hours, and the LC3 was analyzed by immunofluorescent staining. The results are representative blot images of three independent experiments in A, B, D, F, G, I, J, respectively.

As mentioned above, lycopene enhanced the activity of Nrf2 significantly. To unravel the mechanism by which lycopene mediated Nrf2 activation, we firstly studied whether MAPK and Akt, as kinases that can regulate this pathway and can be phosphorylated by lycopene, participated in this regulatory process [35, 36]. As presented in Figure 7A–7C, lycopene-induced Nrf2 translocation in JB6 P+ cells is not affected by the pharmacological inhibition of either kinase. Dissimilarly, western blot analysis revealed lycopene treatment resulted in a significant increase in the total Nrf2 level (Figure 7D). In addition, we focused on the translational and post-translational regulation of Nrf2 which was evidenced by our observation that lycopene did not alter the mRNA level of Nrf2 (Figure 7E). After cycloheximide (CHX) treatment to block protein synthesis, the Nrf2 levels with or without lycopene were measured over time. As shown in Figure 7F, Nrf2 levels decreased by 100% within 12 h in the presence of CHX alone. Nevertheless, further lycopene treatment reduced the level of Nrf2 protein by less than 75% within 12 h, so lycopene caused Nrf2 enhancement following a posttranslational mechanism.

As a key inhibitor of Nrf2, Keap1 is the central regulatory protein at the posttranslational level [37]. Lycopene markedly decreased the level of Keap1 protein both time- and dose-dependently (Figure 7G). Keap1 mRNA levels were detected by RT-PCR to elucidate the steps in which lycopene suppressed Keap1 via the Keap1 biosynthesis pathway (Figure 7H). Since this compound did not influence Keap1 mRNA levels, its regulatory role should exist at the transcriptional or posttranscriptional level. By using CHX, we then proved that lycopene induced Keap1 reduction mainly following a posttranslational mechanism (Figure 7I).

Posttranslational protein degradation mainly occurs via the autophagy-lysosomal pathway and the ubiquitin-proteasomal pathway [38]. To determine the pathway with which lycopene induced Keap1 reduction, cells were first treated by lycopene and/or MG132, a ubiquitination-proteasome inhibitor. Keap1 reduction induced by lycopene, which was barely affected by MG132, was blocked by 3-MA, an autophagy inhibitor (Figure 7J). Since 3-MA can affect many cellular processes besides anti-autophagy, we blocked autophagy by using chloroquine (CQ), an autophagosome-lysosome inhibitor. Likewise, cells treated with CQ showed no Keap1 degradation in response to lycopene treatment either (Figure 7J). To further confirm that the autophagy pathway was involved in Keap1 degradation induced by lycopene, we tested the level of LC3-, representing the number of autophagosomes and indicating activation of the autophagy pathway. Lycopene elevated the LC3 level in dose-dependently manner (Figure 7K). Taken together, lycopene mediated the reduction of Keap1 protein via the autophagy degradation pathway.

### Lycopene facilitated the interaction between p62 and Keap1 that conferred Keap1 degradation accompanied by Nrf2 stabilization and its preventive effects on cutaneous tumor

**Figure 8:**
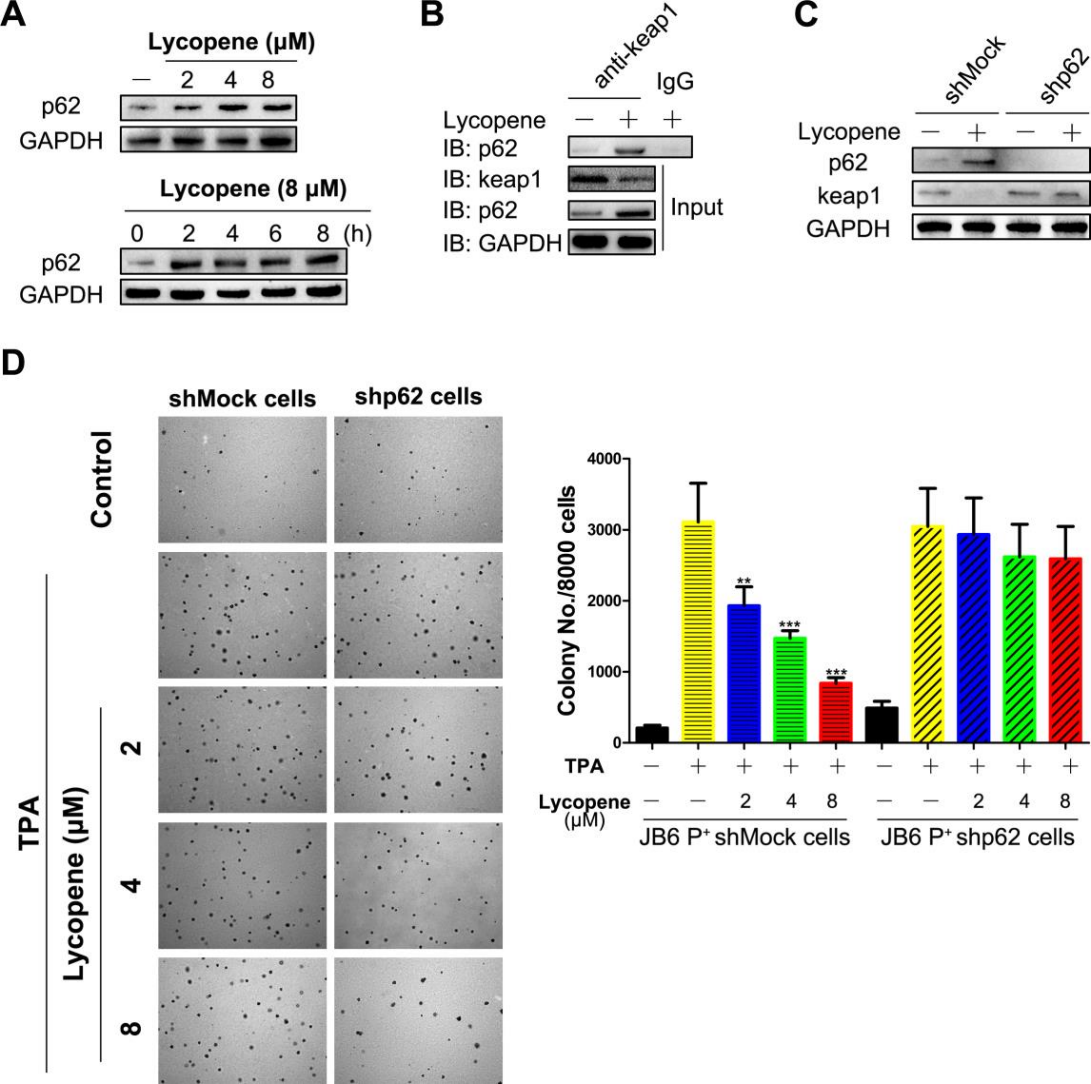
**p62 mediates lycopene-induced keap1 degradation and cutaneous tumor prevention.** (**A**) Time-dependent (bottom) and dose-dependent (top) study of p62 protein levels. Cells were pretreated with 8 μM lycopene for different times or with different doses of lycopene for 6 hours and then exposed with TPA for additional 2 hours, and the p62 was analyzed by western blot. (**B**) Cells were treated with lycopene for 4 hours and then exposed with TPA for additional 1 hours. Co-IP was done as described in Methods, and the immunoprecipitants were immunoblotted using antibodies for keap1, p62 and GAPDH. (**C**) Cells were transfected with shRNA specific for p62 or a nonspecific control shRNA for hours and then treated with lycopene (8 μM) for 6 hours and then TPA for 2 additional hours. Keap1 and p62 proteins were assayed by Western blot. (**D**) Inhibitory effect of lycopene pretreatment on the TPA-induced transformation of shMock- and shp62-transfected JB6 P+ cells. (right) Quantitative analysis of this soft agar assay (n=3). The data are presented as the mean ± SD. **p < 0.01, ***p < 0.001 (versus TPA alone). The results are representative blot image of three independent experiments shown in **A**, **B**, **C**, respectively.

To find a direct autophagy-related mediator, we focused on p62 because elevation in its expression can promote p62/Keap1 binding and can compete for Keap1 binding with Nrf2, contributing to activation of the Nrf2 pathway [39]. Figure 8A showed that lycopene raises p62 protein level both dose- and time-dependently, and almost synchronized with Nrf2 increase. As demonstrated by the co-immunoprecipitation assay, lycopene induced p62 binding to Keap1, so Keap1 degradation was mediated by p62 (Figure 8B). To test the role of p62 in Keap1 degradation, p62 was knocked down by using a p62-specific RNA interfering strategy. Knock-down of p62 reversed Keap1 degradation and Nrf2 induction caused by lycopene, supporting that Keap1 degradation was induced by lycopene via the p62-mediated autophagy pathway (Figure 8C). In JB6-shp62 cells, the inhibitory effects of lycopene on the anchorage-independent growth of JB6 P+ cells induced by TPA were weakened (Figure 8D). Collectively, lycopene prevented carcinogen-induced cutaneous tumors mainly by inducing activation of the Nrf2 pathway through p62-triggered autophagic Keap1 degradation.

## DISCUSSION

Until now, cutaneous carcinoma remains the most common human malignancy (particularly for the white population) owing to increasing urbanization, rising life expectancy, and lifestyle changes. Millions of new cases are being diagnosed worldwide annually. Even if effective treatment modalities, including chemotherapy, radiotherapy, immunotherapy, photodynamic therapy, and selective inhibitors, have been developed successfully, the burden of cutaneous carcinoma is still chronically high [3]. Alternatively, as one of the most promising approaches that suppress, reverse, or block carcinogenesis in the initiation, promotion, or progression phase, chemoprevention has been widely used to decrease the rates of cutaneous carcinoma incidence and related death [5].

Lycopene, the principle phytochemical found in tomato, also a popular additive in skin-care products, has been found to have multiple associated biological functions for cutaneous tissue, including anti-inflammatory, anti-microbial, and anti-aging effects. For the first time, we herein found in a DMBA-initiated, TPA-promoted mouse model of cutaneous papilloma that lycopene obviously decreased cancer incidence rate and multiplicity, delayed the latency, and kept the benign nature of tumors only in the promotion phase through a kind of grouping unlike most previous studies. Additionally, most currently available preclinical studies concerning natural compounds were performed by using fully transformed cancer cells in vitro. In this study, we also verified that lycopene prevented the carcinogenesis of pre-malignant JB6 P+ cells in vitro by inhibiting TPA-stimulated malignant transformation. As suggested by the significant reduction of chemically induced tumorigenesis of cutaneous tissues and cells, lycopene may be an eligible cancer chemopreventive agent. Therefore, the mechanisms by which lycopene exerted antitumor effects in vitro and in vivo should be further explored.

By using a comprehensive strategy, we predicted that lycopene prevented mouse cutaneous tumors in the promotion phase with association of intracellular autophagy and redox state. As the tumor promoter for the model we used, TPA evidently stimulated the malignant transformation of preneoplastic cells by disruption of redox balance, which was significantly inhibited by lycopene pretreatment both in vitro and in vivo. Furthermore, the antioxidant enzyme defense system was also enhanced by lycopene, suggesting that lycopene exerted preventive effect on cutaneous tumor by keeping intracellular redox homeostasis.

The transcriptional activation of gene-encoding cytoprotective or antioxidant proteins was dominantly regulated by the redox-sensitive transcription factor Nrf2. Lycopene activated the transcription and nuclear translocation of Nrf2, and induced the expression of downstream genes. Interestingly, this effect can be exponentially enhanced with TPA present; suggesting the a primary ability of lycopene to be that of maintaining equilibrium against intracellular stressors caused by carcinogens rather than that of normal cells, and this action model of drugs may lead to a relatively lower adverse reaction. In addition, Nrf2 was crucial for lycopene to maintain cellular redox homeostasis and to relieve tumorigenesis in both gene knock-down cells and transgenic mice.

Lycopene pretreatment induced Nrf2 protein accumulation, but did not change its mRNA level. Nrf2 is a stress-responsive transcription factor that is constantly degraded by the Keap1-Cul3 E3 ubiquitin ligase complex through polyubiquitination [34]. Upon stress challenges, Nrf2 escapes polyubiquitination by this complex and accumulates in the cell nucleus, inducing the transcription of stress-responsive genes. Nrf2 activation by cancer chemopreventive agents has been explained by two plausible mechanisms. One is Keap1 inhibition, and the other is Nrf2 phosphorylation by kinases such as Akt and MAPKs [37]. To clarify the mechanisms underlying lycopene-induced Nrf2 activation, we first focused on the upstream kinases well-documented to participate in the activation of Nrf2 signaling. Lycopene induced the phosphorylation of p38 MAPK and ERK, but blocking these two kinases did not affect nuclear Nrf2 localization, implying that the second mechanism may not be applicable to this study. Based on this, we studied the plausibility of the first mechanism. It is well-established that endogenous Keap1 level plays an important role in controlling Nrf2 activity, and that knock-down of Keap1 gene can boost Nrf2 activity and alleviate oxidative stress and steatosis induced by fasting [37]. Lycopene herein induced Keap1 reduction at the protein level, because its mRNA level remained constant. To the best of our knowledge, microbial and cellular components are eliminated mainly by autophagy and ubiquitination to maintain homeostasis. In this study, autophagy mediated the inhibitory effects of lycopene on Keap1 protein. First of all, MG132, a proteasome inhibitor, did not work, but 3-MA and CQ, as specific inhibitors for autophagy, abolished lycopene-induced reduction of Keap1 protein. Secondly, lycopene treatment increased LC3 and LC3-II cleavage and synchronized to the Keap1 degradation.

We herein demonstrated that pretreatment with lycopene induced autophagy by raising p62 protein level and enhancing the binding of p62 to Keap1. In the absence of p62, lycopene did not induce Keap1 degradation. Similarly, knock-down of p62 reversed the inhibitory effects of lycopene on the malignant transformation of JB6 P+ cells induced by TPA, indicating that lycopene prevented cutaneous tumors through inducing the Nrf2 pathway activation by p62-triggered autophagic Keap1 degradation.

**Figure 9:**
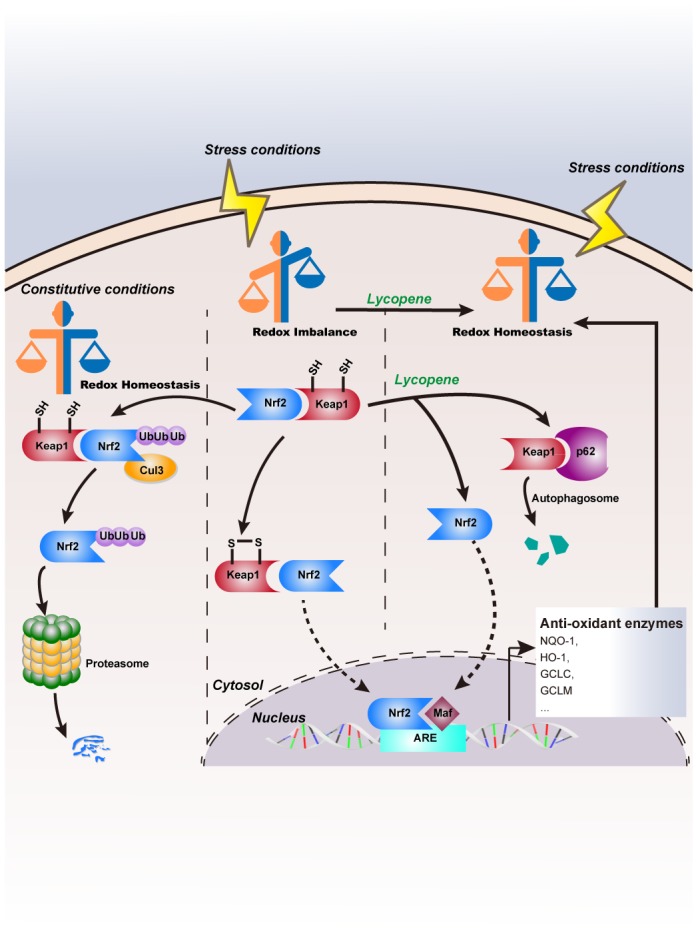
**Schematic model of lycopene’s inhibitory effects on cutaneous tumor progression.**

In summary (Figure 9), topical lycopene application inhibited TPA-induced intracellular redox imbalance and mouse cutaneous tumors in the promotion phase by accelerating nuclear localization of Nrf2, which may be mediated by up-regulating p62 protein levels, facilitating Keap1 degradation in an autophagy-lysosomal pathway. Based on the results of this study, lycopene or tomato-related skin-care products in combination with protective sun screen may be helpful in reducing skin cancer in humans.

## MATERIALS AND METHODS

### Chemicals and reagents

Lycopene (Catalog NO. SMB00706-5MG) from sigma for *in vitro* cell model studies. Lycopene (Catalog NO.1370860-500MG) from Sigma for *in vivo* mouse model studies. 7,12-dimethylbenzanthracene (DMBA), cycloheximide (CHX), MG132, chloroquine (CQ), and 3- methyladenine (3-MA) were purchased from Sigma-Aldrich Chemical Co. (St. Louis, USA). 12-O-tetradecanoyl-phorbol 13-acetate (TPA) was obtained from Cayman Chemical Company (Michigan, USA). Fetal bovine serum (FBS), minimum essential medium (MEM), and trypsin-EDTA solution were purchased from Gibco Laboratories. U0126 and SB203580 were bought from Selleck, USA.

### Cell line and cell culture

The mouse epidermal cell line, JB6 P+ (JB6 Cl 41-5a), from American Type Culture Collection (ATCC) were maintained in MEM containing 10% FBS in a humidified 5% CO2 atmosphere at 37°C. The JB6 P+ epidermal cells are derived from mouse skin and are regarded as an appropriate cell model for studying the chemopreventive effect and underling mechanisms of lycopene in vitro.

### Establishment of carcinogenesis model induced by DMBA/TPA

Female Institute of Cancer Research (ICR) mice aged 6–7 weeks were supplied from Beijing Vital River Laboratory Animal Technology Co., Ltd and housed in climate-controlled quarters with a 12-h light/12-h dark cycle. All experimental procedures were carried out in accordance with the Guide for the Care and Use of Laboratory Animals, and before the animal experiments were carried out, the procedures were approved by the Research Ethical Committee of Nanjing University of Chinese Medicine. ICR mice were randomly divided into five groups, 10 animals per group. The workflow and animal grouping of the in vivo study was depicted in Figure 1A. Specifically, mouse in all the groups were subjected to DMBA (60 μg) dissolved in 0.2 mL topically on the naked backs. The first week after tumor initiation with DMBA, animals were further exposed to TPA (4 μg) twice a week for a total of 32 weeks: Model group (M). Group A (Acetone group) was the vehicle control group. Mice treated with lycopene (8 μmol in 0.2 mL of acetone) were topically applied five times a week with different initiations and durations designed in Figure 1A. Tumors with more than 1 mm diameter were counted every week. Nrf2-/- mice were gifted by Prof. Peng Cao from Jiangsu Province Academy of Chinese Medicine.

### Histological assessment

After the animals were sacrificed, the skin tissue was isolated and part of the fresh tissues were fixed in 4% paraformaldehyde and sent for hematoxylin and eosin (H&E) staining. Sections were photographed using the ZEN 2011 imaging software on a Zeiss invert microscope under 40-fold magnification.

### Measurement of 8-OhdG, 4-NHE, ROS, GSH/GSSG and antioxidant enzymes activity in tissues

Part of the fresh skin tissues were snap frozen in liquid nitrogen after excision for further process. Measurement was performed using the commercial kits according to manual instructions. The reduced glutathione and oxidized glutathione (GSH/ GSSG) Quantification Kit, reactive oxygen species (ROS) assay kit, catalase (CAT) activity assay kit, glutathione peroxidase (GPx) assay kit, superoxide dismutase (SOD) assay kit, and glutathione reductase (GR) assay kit were procured from Beyotime, China. The 4-hydroxy-2-nonenal (4-HNE) ELISA kit and 8-hydroxy-2’-deoxyguanosine (8-OhdG) ELISA kit were from Cell Biolabs, USA.

### Protein isolation and western blot analysis

Protein lysates of cells or tissue were prepared with RIPA lysis buffer containing protease and phosphatase inhibitors. Nuclear and cytoplasmic cell extracts were prepared using the NE-PER Nuclear and Cytoplasmic Extraction kit (Thermo). Equal amounts of protein lysates (50 μg) were loaded on SDS-PAGE and transferred onto PVDF membranes. After membranes were blocked with 5% skimmed milk at room temperature for 2-3 hours, they were incubated with antibody against Nrf2 (1: 1000, Abcam, Cat.NO. ab137550), p62 (1: 1000, bioworld, Cat.NO. AP6006), Keap1 (1:500, Santa Cruz, Cat.NO. sc-33569), LaminB1 (1: 1000, Abcam, Cat.NO. ab133741), GAPDH (1: 6000, Bioworld Technology, Cat.NO.AP0063), HO-1 (1: 1000, Abclonal, Cat.NO. A1346), and NQO1 (1:1000, Abclonal, Cat.NO. A0047) followed by incubation with goat anti-rabbit IgGs -HRP (1: 10000, Bioworld Biotechnology, Cat.NO. BS 13278). Target proteins were detected by the ECL system (Millipore) and visualized with the ChemiDoc XRS system (Bio-Rad).

### Immunohistochemical (IHC) staining

For IHC analysis of Nrf2 protein, mice tissue samples were collected and paraformaldehyde fixed, and paraffin-embedded sections of skin tissues (4 μm thick) were mounted on slides coated with 2-aminopropyltriethoxysilane, baked, deparaffinized, and rinsed with 3% hydrogen peroxide, and then incubated with proteinase K (0.5 mg/mL). After that, these sections were washed and then blocked with StartingBlockTM blocking buffers (Pierce, Rockford, IL, USA) for 5 min and subsequently incubated with an anti-Nrf2 (1: 100, Abcam, Cat.NO. ab137550) polyclonal antibody for 30 min. Finally, the sections were incubated with Streptavidin-Biotin Complex (Solarbio) for 30 min at room temperature, followed by detection with a 3,3-diaminobenzidine tetrahydrochoride solution (chromogen) (ZSGB-BIO) and counterstained with hematoxylin (Solarbio, China). Sections were further mounted with neutral gums. IHC sections were photographed by Mantra 1.01(Perkin Elmer).

### Immunofluorescent staining

After JB6 P+ cells seeded and grown on glass cover slips were treated by indicated agents, they were fixed by pre-cold acetone, then rinsed three times with 1× phosphate-buffered saline (PBS). The cells were permeabilized in 0.1% Triton X-100 and incubated with 1% bovine serum albumin (BSA) in 1× PBS to block nonspecific binding. Subsequently, the cells were immunostained by incubating with Nrf2 antibody (1: 100, Abcam, Cat.NO. ab137550) overnight at 4°C. After being washed with PBS, cells were incubated with Rhodamin-conjugated goat anti-rabbit antibody (1: 200, CWBIO, China, Cat.NO. BA1105). Actin filaments were stained using Actin-Tracker Green (Beyotime, China). Nuclei were counterstained with Hoechst 33258 (Beyotime, China). Fluorescent images were taken and analyzed using the ZEN pro 2012 imaging software on a Zeiss invert microscope under 200-fold magnification.

### Co-immunoprecipitation assay

For co-immunoprecipitation experiment, cell lysates adjusted to 1 mg/mL protein were precleared by antibody keap1 (1: 20, Santa Cruz, Cat.NO. sc-33569). After gentle rocking at 4°C overnight, Protein A/G PLUS-Agarose (Santa Cruz, Cat.NO. sc-2003) was added to the lysate/antibody mixture, and incubated with gentle agitation at 4°C for 4 h. Then the immunoprecipitates were collected by centrifugation (12000 rpm, 4 min) and washed three times with cell lysis buffer (NP-40, Beyotime), then boiled for 5 min with the same volume of 2× loading buffer (62.5 mM Tris-HCl, pH 6.8, 2% w/v SDS, 10% glycerol, 50 mM DTT, 0.01% w/v bromophenol blue). Protein interactions were analyzed via Immunoblot for p62.

### RNA isolation and quantitative real-time PCR

Total RNA was extracted from the tissue samples or cells using the TRIzol reagent (Invitrogen). First-strand cDNA was synthesized with 500 ng total RNA using a Hiscript® II QRTSuperMix (Vazyme, China). Quantitative RT-PCR was performed using the SYBR Green Master kit (Bio-Rad, USA) according to the manufacturer’s instructions. The comparative cycle threshold (Ct, 2(-ΔΔCt)) method was applied to quantify the relative gene expression levels. The primers used for qRT-PCR were as follows: gapdh: 5’-GGTTGTCTCCTGCGACTTCA-3’ (forward) and 5’-TGGTCCAGGGTTTCTTA CTCC-3’ (reverse); cat: 5’-CCCCTATTGCCGTTCGATTCT-3’ (forward) and 5’-TTCAGGTGAGTCTGTGGGTTT-3’ (reverse); sod1: 5’-AACCAGTTGTGTTG TCAGGAC-3’ (forward) and 5’-CCACCATGTTTCTTAGAGTGAGG-3’ (reverse); gsr: 5’-GCGTGAATGTTGGATGTGTACC-3’ (forward) and 5’-GTTGC ATAGCCGTGGATAATTTC-3’ (reverse); gpx1: 5’-AGTCCACCGTGTATGCCTTCT-3’ (forward) and 5’-GAGACGCGACATTCTCAATGA-3’ (reverse); gclc: 5’-GGGGTGACGAGGTGGAGTA-3’ (forward) and 5’-GTTGGGGTTTGTCCTCTC CC-3’ (reverse); gclm: 5’-AGGAGCTTCGGGACTGTATCC-3’ (forward) and 5’-GGGACATGGTGCATTCCAAAA-3’ (reverse); hmox1: 5’-CACGCATA TACCCGCTACCT-3’ (forward) and 5’-CCAGAGTGTTCATTCGAGCA-3’ (reverse); nqo1: 5’-TTCTCTGGCCGATTCAGAGT-3’ (forward) and 5’-GGCTGCT TGGAGCAAAATAG-3’ (reverse); nfe2l2: 5’-CTCGCTGGAAAAAGAAGTGG-3’ (forward) and 5’-CCGTCCAGGAGTTCAGAGAG-3’ (reverse); keap1: 5’-TGCCCCTGTGGTCAAAGTG-3’ (forward) and 5’GGTTCGGTTACC GTCCTGC-3’ (reverse).

### shRNA knockdown

Predesigned Nrf2-knockdown shRNA construct and p62-knockdown shRNA construct were purchased from Sigma-Aldrich with Catalog NO. SHCLNG-NM_010902_TRCN0000054658 and SHCLNG-NM_011018 _TRCN0000098619, respectively. Vehicle control construct was also provided from Sigma-Aldrich. The sequences for the mouse NRF2-shRNA are CCGGCCAAAGCTAGTATAGCAATAACTCGAGTTATTGCTATACTAGCTTTGGTTTTTG. The sequences for the mouse p62-shRNA are CCGGGAGGTTGACATTGATGTGGAACTCGAGTTCCACATCAATGTCAACCTCTTTTTG. The plasmid was transfected using lipofectamine 2000 according to manual’s instructions.

### Data collection and analysis of target genes related to the promotion phase of cutaneous carcinoma

Microarray data with accession number E-MEXP-188 was downloaded from the Arrayexpress database (http://www.ebi.ac.uk/arrayexpress/) [27]. In brief, one set of comparison were conducted. DMBA-initiated skin (D) was compared with DMBA-initiated, TPA-promoted skin (DT). Different expression genes (DEGs) were defined, and P< 0.05, Fold Chang (FC) > 1.5 was considered as the cutoff value.

### Protein-protein interaction (PPI) network construction

PPI data was imported from six currently available PPI databases including the Biological General Repository for Interaction Datasets (BioGRID: https://thebiogrid.org), the Biomolecular Interaction Network Database (BIND: http://binddb.org), the Molecular Interaction Database (MINT: http://mint.bio.uniroma2.it/mint/), the Human Protein Reference Database (HPRD: http://www.hprd.org), the Database of Interacting Proteins (DIP: http://dip.doe-mbi.ucla.edu/dip/Main.cgi), and the Biological General Repository for Interaction Datasets by BisoGenet, a Cytoscape plugin [40].

### Definition of topological feature set for the network

By calculating six measures (i.e. degree centrality (DC), betweenness centrality (BC), closeness centrality (CC), eigenvector centrality (EC), network centrality (NC), and local average connectivity(LAC)) with CytoNCA, a Cytoscape plugin, the topological property of every node in the interaction network was analyzed [29]. The definitions and computation equations of these six parameters represent the topological importance of a node in the network. The higher the quantitative values of these parameters, the more important the node in this network.

### Prediction of drug targets for lycopene

The potential targets of lycopene were predicted using PharmMapper server (provided by the Shanghai Institute of Materia Medica, Chinese Academy of Sciences) [26], a web server for potential drug targets identification using pharmacophore mapping approach at http://59.78.96.61/pharmmapper. Briefly, 3D Mol2 file of lycopene (PubChem CID: 12305761) was submitted to PharmMapper server. During the procedure, the maximum conformations were set up to 300, and the number of reserved matched targets was 1000. Other parameters were kept as default. The submission ID can be stored and used to check the prediction results.

### Enrichment analysis

As a Cytoscape plugin visualizing the non-redundant biological terms for large gene clusters in a functionally grouped network, ClueGO was utilized to visualize the enrichment of lycopene candidate targets [30]. The ClueGO network was created by using kappa statistics, reflecting the relationships between the terms on the basis of the similarity between their associated genes. The significances of the terms and groups were calculated automatically.

### Statistical analysis

Values were expressed as the mean ± SD of at least three independent experiments. One way analysis of variance (ANOVA) was used to compare in groups and p < 0.05 was considered as statistically significant.

## Supplementary Material

Supplementary Figure 1

Supplementary Tables
