# Supplementary material for: Lycopene prevents carcinogen-induced cutaneous tumor by enhancing activation of the Nrf2 pathway through p62-triggered autophagic Keap1 degradation
Source: Aging (Albany NY). 2020 May 4;12(9):8167–90. doi: 10.18632/aging.103132 (PMC7244072; doi:10.18632/aging.103132)
Supplement: Supplementary Figure 1 [file aging-12-103132-s001..pdf]

SUPPLEMENTARY FIGURE

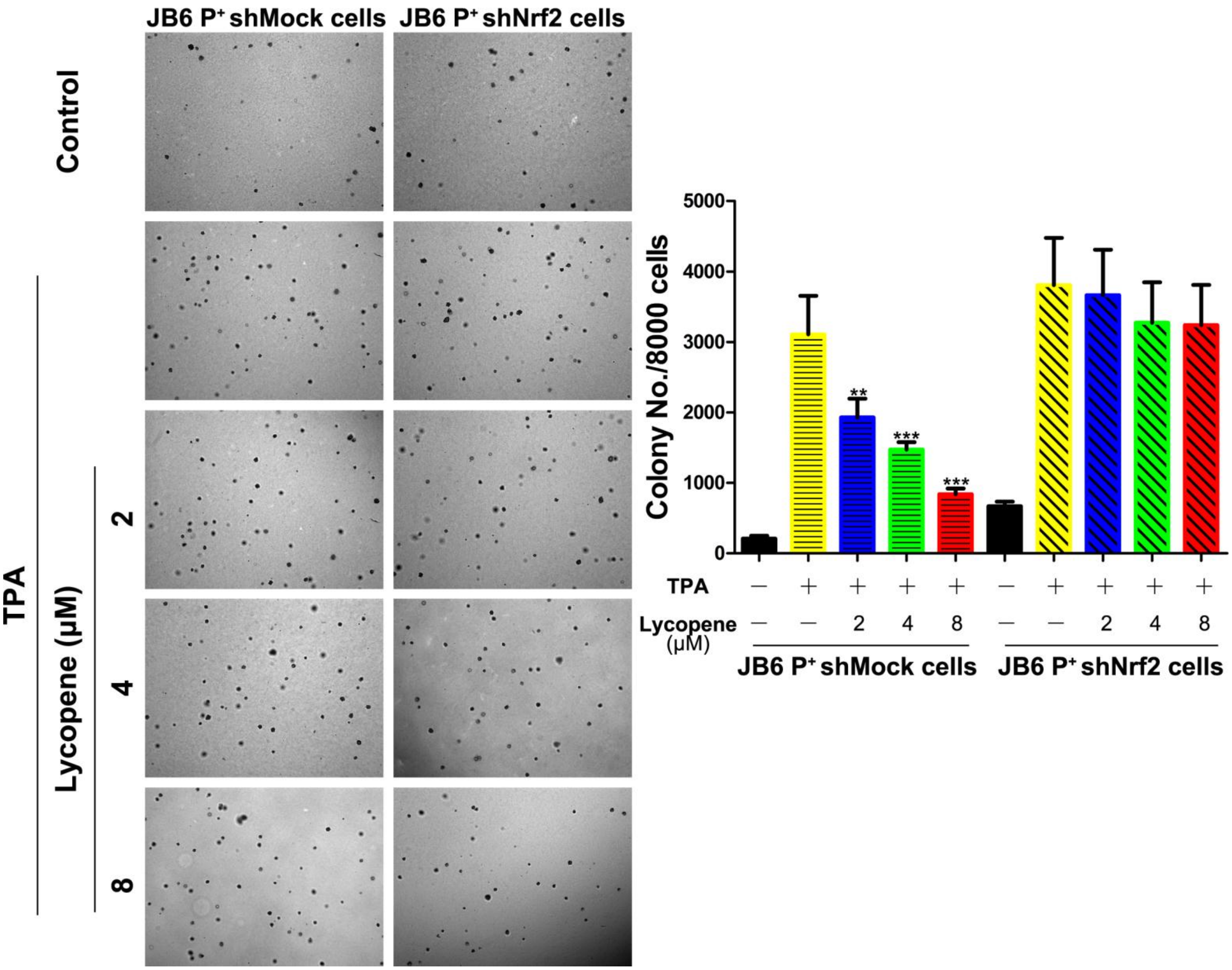

Supplementary Figure 1. Inhibitory effect of lycopene pretreatment on the TPA-induced transformation of shMock- and shNrf2-transfected JB6 P$^+$ cells. Cells (1×10^5/10-cm dish) were treated with lycopene (0–8μM) for 5 days. The pretreated cells (at a density of 8,000 cells/well) were then transferred to soft agar containing TPA in 6-well plates for an additional 2 weeks. The colonies exhibiting anchorage-independent growth were taken and analyzed using the ZEN pro 2012 imaging software on a Zeiss invert microscope under 100-fold magnification. (right) Quantitative analysis of this soft agar assay (n=3). The data are presented as the mean ± SD. $^{**}p < 0.01$, $^{***}p < 0.001$ (versus TPA alone).
