# Supplementary material for: Lycopene prevents carcinogen-induced cutaneous tumor by enhancing activation of the Nrf2 pathway through p62-triggered autophagic Keap1 degradation
Source: Aging (Albany NY). 2020 May 4;12(9):8167–90. doi: 10.18632/aging.103132 (PMC7244072; doi:10.18632/aging.103132)
Supplement: Supplementary Tables [file aging-12-103132-s002..pdf]

SUPPLEMENTARY TABLES

Supplementary Table 1. Putative targets of lycopene.

Target name	Targets Gene name	Uniprot ID
GTPase Hras	HRAS	P01112
Inositol monophosphatase1	IMPA1	P29218
Adenosine kinase	ADK	P55263
Beta-hexosaminidase beta chain	HEXB	P07686
Uridine-cytidine kinase 2	UCK2	Q9BZX2
Hexokinase-1	HK1	P19367
Heat shock protein HSP 90-alpha	HSP90AA1	P07900
histone mRNA exonuclease 1	ERI1	Q8IV48
Protein-glutamine gamma-glutamyltransferase E	TGM3	Q08188
Deoxycytidine kinase	DCK	P27707
Methionine aminopeptidase 2	METAP2	P50579
Proto-oncogene tyrosine-protein kinase LCK	LCK	P06239
Leukocyte elastase	ELANE	P08246
Ephrin type-A receptor 2	EPHA2	P29317
Cathepsin K	CTSK	P43235
Nitric oxide synthase, inducible	NOS2	P35228
Death-associated protein kinase 1	DAPK1	P53355
Prothrombin	F2	P00734
Hypoxanthine-guanine phosphoribosyltransferase	HPRT1	P00492
Histo-blood group ABO system transferase	ABO	P16442
Glutathione S-transferase P	GSTP1	P09211
Vitamin D3 receptor	VDR	P11473
E-selectin	SELE	P16581
Lanosterol synthase	LSS	P48449
Tyrosine-protein phosphatase non-receptor type 1	PTPN1	P18031
ADP-ribosylation factor-like protein 5B	ARL5B	Q96KC2
Mitogen-activated protein kinase 10	MAPK10	P53779
Dipeptidase 1	DPEP1	P16444
Estradiol 17-beta-dehydrogenase 1	HSD17B1	P14061
UDP-N-acetylhexosamine pyrophosphorylase	UAP1	Q16222
Sulfotransferase family cytosolic 2B member 1	SULT2B1	O00204
Thymidylate kinase	DTYMK	P23919
Aldo-keto reductase family 1 member C2	AKR1C2	P52895
GTP-binding nuclear protein Ran	RAN	P62826
Riboflavin kinase	RFK	Q969G6
S-methyl-5-thioadenosine phosphorylase	MTAP	Q13126
Mitogen-activated protein kinase 14	MAPK14	Q16539
Beta-secretase 1	BACE1	P56817
3-phosphoinositide-dependent protein kinase 1	PDPK1	O15530
Serine/threonine-protein kinase 6	AURKA	O14965
Chitotriosidase-1	CHIT1	Q13231
Phenylethanolamine N-methyltransferase	PNMT	P11086
Cell division protein kinase 2	CDK2	P24941
Proto-oncogene tyrosine-protein kinase Src	SRC	P12931
Tryptophan 5-hydroxylase 1	TPH1	P17752
Early endosome antigen 1	EEA1	Q15075
5(3)-deoxyribonucleotidase, mitochondrial	NT5M	Q9NPB1
Glucose-6-phosphate isomerase	GPI	P06744
Ras-related protein Rap-2a	RAP2A	P10114
Glycogen phosphorylase, liver form	PYGL	P06737
Apoptotic protease-activating factor 1	APAF1	O14727
Corticosteroid 11-beta-dehydrogenase isozyme 1	HSD11B1	P28845

Androgen receptor	AR	P10275
Glutathione S-transferase Mu 2	GSTM2	P28161
Glutathione S-transferase A3	GSTA3	Q16772
cAMP-specific 3,5-cyclic phosphodiesterase 4D	PDE4D	Q08499
Carbonic anhydrase 2	CA2	P00918
Deoxyuridine 5-triphosphate nucleotidohydrolase, mitochondrial	DUT	P33316
Histamine N-methyltransferase	HNMT	P50135
Renin	REN	P00797
Nuclear factor erythroid 2-related factor 2	NFE2L2	Q16236
Tryptophanyl-tRNA synthetase, cytoplasmic	WARS	P23381
Dual specificity mitogen-activated protein kinase kinase 1	MAP2K1	Q02750
Serum albumin	ALB	P02768
Cyclin-A2	CCNA2	P20248
Neutrophil collagenase	MMP8	P22894
cAMP-dependent protein kinase catalytic subunit alpha	PRKACA	P00517
Glucocorticoid receptor	NR3C1	P04150
ADP-ribosyl cyclase 2	BST1	Q10588
Baculoviral IAP repeat-containing protein 7	BIRC7	Q96CA5
Maleylacetoacetate isomerase	GSTZ1	O43708
Glucosylceramidase	GBA	P04062
Estrogen recepto	ESR1	P03372
Inosine-5-monophosphate dehydrogenase 2	IMPDH2	P12268
Protein S100-A9	S100A9	P06702
Alcohol dehydrogenase class-3	ADH5	P11766
Peptidyl-prolyl cis-trans isomerase FKBP1A	FKBP1A	P62942
tRNA (cytosine-5-)-methyltransferase	TRDMT1	O14717
Histidine triad nucleotide-binding protein 1	HINT1	P49773
Mast/stem cell growth factor recepto	KIT	P10721
Sepiapterin reductase	SPR	P35270
Caspase-1	CASP1	P29466
Ferrochelatase, mitochondrial	FECH	P22830
Tyrosine-protein kinase JAK2	JAK2	O60674
Aldose reductase	AKR1B1	P15121
Thymidylate synthase	TYMS	P04818
Sex hormone-binding globulin	SHBG	P04278
Amine oxidase [flavin-containing] B	MAOB	P27338
Fibrinogen gamma chain	FGG	P02679
Tyrosine-protein kinase HCK	HCK	P08631
Glutathione S-transferase theta-2	GSTT2B	P0CG30
NAD-dependent deacetylase sirtuin-5	SIRT5	Q9NXA8
Glutathione S-transferase A1	GSTA1	P08263
Proto-oncogene serine/threonine-protein kinase Pim-1	PIM1	P11309
Estrogen receptor beta	ESR2	Q92731
Insulin receptor	INSR	P06213
RAC-beta serine/threonine-protein kinase	AKT2	P31751
Serine/threonine-protein phosphatase PP1-gamma catalytic subunit	PPP1CC	P36873
Mitogen-activated protein kinase 1	MAPK1	P28482
Glycogen synthase kinase-3 beta	GSK3B	P49841

Supplementary Table 2. Topological feature values of targets for Lycopene preventing cutaneous tumor.

Candidate targets GENE NAME	DC(>111)	EC(>0.039842676)	LAC(>24.66197183)	BC(>1606.42844)	CC(>0.339340764)	NC(>36.7491238)
ABCC2	187	0.045459241	24.84408602	9005.802877	0.347414421	68.46314328
ABCC4	305	0.094544813	38.28618421	13699.7453	0.35775	130.7524166
ATG12	325	0.093174718	30.97222222	12510.34332	0.358556753	120.1888415
ATG3	170	0.045113869	32.04142012	5216.234315	0.345318533	68.56396477
ATG4	118	0.040851254	26.58974359	1696.036371	0.340309156	39.22334829
ATG5	136	0.047911584	24.88888889	3248.988206	0.342344498	38.8965772
BCL2	216	0.069759451	31.7627907	4482.16925	0.348005837	74.56607043
BECN1	132	0.050224762	28.71755725	2447.53254	0.341201717	38.96404899
BRCA1	229	0.061842397	29.9122807	9281.742982	0.350649351	83.11698941
CAND1	257	0.088335834	44.3671875	5848.056945	0.351856405	121.112482
CAT	297	0.095324956	42.10472973	9978.227127	0.356058721	134.0250891
UGT1A6	401	0.101440765	31.1675	34118.21889	0.365984655	171.5034149
CUL3	407	0.115885355	40.88423645	24462.79402	0.367017184	197.2527924
CYP1A1	205	0.06863936	29.61764706	4398.16936	0.34657302	66.2786147
E2F1	338	0.099740505	34.33234421	18813.26649	0.359909457	134.140268
CES1R	289	0.061022762	25.23958333	22322.9337	0.35667996	113.2022051
TNFSF15	341	0.090039417	34.64705882	25872.36959	0.361363636	153.0423094
GABARAP	140	0.058590103	35.47482014	1898.237411	0.342754491	48.34950655
GCLC	147	0.0477276	34.95205479	2191.891671	0.341935484	58.74549889
GCLM	142	0.051684093	31.26950355	1895.099055	0.341527446	49.3583818
GPX1	183	0.065052092	33.65934066	3556.437636	0.34457019	64.67704418
GRB2	241	0.058540106	25.25	17419.63786	0.352116142	89.14903859
GSTA2	142	0.056025203	34.65957447	1951.962231	0.341772152	51.10983269
GSTM3	210	0.063890435	27.15789474	5601.380232	0.347414421	65.22987287
GSTT1	174	0.063643008	36.75722543	4759.911411	0.345735685	64.08725927
HDAC1	198	0.05421263	35.39593909	6851.173308	0.348090489	88.68155071
HMOX1	226	0.05821839	34.08888889	9092.883339	0.350735294	96.94870092
HNRNPM	141	0.056462422	39.79432624	1662.939308	0.341772152	58.86623363
HNRNPU	174	0.065748565	38.20114943	3281.265977	0.345401883	69.09646809
HSP90AA1	339	0.082687452	33.62426036	31931.63971	0.361546235	164.7935414
HSP90AB1	267	0.070742942	29.7406015	17922.35124	0.35464684	109.7798907
HSPA1A	132	0.048693269	27.44274809	1864.405051	0.342017208	40.24812122
HSPA1B	132	0.048693269	27.44274809	1864.405051	0.342017208	40.24812122
HSPA5	160	0.058036871	29.9	3468.738646	0.344819277	50.6036435
HSPA8	180	0.062471829	32.83240223	5918.755949	0.346825012	59.95911373
ILF3	137	0.056275345	40.18978102	2331.045394	0.341039085	55.95388237
ITGA4	270	0.083415017	31.17472119	7562.414094	0.35368265	92.31751939
KEAP1	128	0.048658811	26.13385827	3073.985256	0.340309156	37.50242308
MAPK14	187	0.051795304	25.40322581	6723.270842	0.346825012	59.11954833
MAPK8	136	0.042939164	25.40740741	2383.545205	0.341364504	37.29512941
RXRA	224	0.058828417	24.86995516	8653.855305	0.350735294	69.8637354
NEDD8	303	0.093732998	39.20198675	12086.99334	0.357214179	130.9642558
NPM1	204	0.076828629	43.01477833	4349.055097	0.348599269	80.91094074
NQO1	171	0.064798295	36.90588235	2511.443348	0.345401883	63.25168001
OBSL1	274	0.080773741	28.08791209	7788.298099	0.353420598	92.19721579

PAN2	177	0.06097826	26.20454545	2525.552876	0.343659942	49.2803812
PARP1	157	0.054239359	32.69230769	3737.515667	0.344238634	54.62459615
PIK3C3	144	0.047793798	25.74125874	2406.579138	0.342754491	40.70780914
PRKDC	149	0.05278014	27.99324324	3848.36108	0.344155844	44.66920169
RNF2	261	0.07375174	29.17307692	9571.492768	0.352463054	92.0338035
RPA1	168	0.055030849	28.65868263	3530.430959	0.344238634	54.14086487
RPS27A	149	0.04911413	30.58108108	3400.264724	0.342754491	52.10114428
SLC2A5	123	0.044492684	26.1147541	1714.945766	0.339501779	38.54230868
SLC6A15	256	0.080202766	28.19215686	5862.321258	0.351942941	79.89887905
SMARCA4	132	0.042924434	31.64885496	2428.652733	0.340552118	50.2666139
SOD2	526	0.129363835	38.59047619	55258.12593	0.378471304	274.9394148
SQSTM1	132	0.047083173	25.96183206	2480.463799	0.341853798	39.09137158
STAU1	168	0.05662981	27.60479042	2627.715126	0.343659942	50.88887432
SUMO1	354	0.096740618	35.40509915	21643.43319	0.361911988	150.7748874
SUMO3	325	0.096635871	35.95061728	15656.75364	0.358466934	134.749212
SUZ12	166	0.048357066	25.02424242	3604.252635	0.342918763	48.82167063
TARDBP	161	0.058899906	37.3	2500.246986	0.343083193	64.12700197
TP53	389	0.105065025	40.52835052	31882.01865	0.365797546	190.5462544
TUBB	131	0.049730685	26.50381679	1763.81893	0.342098972	37.64882082
TXNRD1	339	0.102408722	43.83727811	20720.81764	0.361454913	164.0209297
UBC	1310	0.239188775	56.85026738	795175.8572	0.479557641	1310.861462
UBL4A	139	0.048587617	30.18115942	1804.806801	0.340471092	46.31340527
UGT1A1	176	0.0592997	33.22857143	4093.978998	0.345485273	62.79719772
ULK1	197	0.062763587	31.25510204	5685.021811	0.347667638	68.03153982
VCP	213	0.065383755	31.54716981	8173.289899	0.349621305	76.80972806
YWHAG	173	0.051689923	27.61046512	4841.588854	0.345902828	58.95030038
